# Retraction: Upregulation of SATB1 Is Associated with Prostate Cancer Aggressiveness and Disease Progression

**DOI:** 10.1371/journal.pone.0344761

**Published:** 2026-03-12

**Authors:** The PLOS One Editors

After this article [1] was published, concerns were raised about the reported mouse tumor sizes, mouse body weight, results presented in Fig 5, and the statistical analyses of the data reported in Fig 6. Specifically:

- The tumor burden reported in [1] exceed standards commonly accepted for mouse tumor studies. Editorial assessment of the individual-level underlying data for Figs 6A and 6C provided by the corresponding author raises concerns that prior to the final time-point (day 31):
  - Individual tumor sizes exceeded 2,000 mm^3^ (up to 2,738 mm^3^) in 25% (2 out of 8) of the mice;
  - Weight loss (calculated using body weight excluding tumor mass) exceeded 20% in 37.5% (3 out of 8) of the mice.
- Predefined humane endpoints, method of euthanasia, and the ethics approval document number were not reported in [1].
- In the Fig 5A E-cadherin panel, there appear to be horizontal discontinuities between lanes 4 and 5, as well as between lanes 6 and 7.
- In Fig 5C, the PZ-HPV-SATB1 Migration panel partially overlaps with the PZ-HPV-SATB1 Invasion panel.
- In Figs 6A and 6C, a statistically significant reduction in tumor growth with a decrease in tumor weight and volume was reported (*P* < 0.003). However, the statistical tests described in the Materials and Methods section do not account for the lack of independence among observations. In addition, considering the limited sample sizes, assumptions regarding the underlying data distribution are unlikely to be met.

In response to queries about the mouse experiments, the corresponding author provided additional information pertaining to the methods of euthanasia and general animal monitoring, but the authors did not provide pre-defined humane endpoints and scientific or ethical justification for the tumor sizes, and they were unable to provide the ethics approval documentation or the animal health monitoring log for editorial review.

Regarding the concern about Fig 5A, the corresponding author stated that the irregularities are due to differences in background intensity or contrast adjustments made during image processing. The underlying blots provided for editorial review were of too low resolution to resolve the concerns with Fig 5A. The corresponding author stated an error was made during the preparation of Fig 5C and provided a replacement image.

With regard to the statistical analysis of the data shown in Figs 6A and 6C, the corresponding author stated that the data were reassessed by a biostatistician, who identified errors in data calculation and entry. The newly reported *P* values for Figs 6A and 6C are 0.068 and 0.272, respectively. In light of the statistical reanalysis, the findings in [1] that injection of SATB1 KO clone resulted in reduced tumor growth in mice with a decrease in tumor weight and volume are no longer supported.

The *PLOS One* Editors retract this article in light of the statistical concerns which call into question the validity and reliability of the reported results, and the reported tumor sizes which raise concerns about animal welfare considerations in the study design.

SS, PF, DD, and SG did not agree with the retraction. HS, AA, and GTM either did not respond directly or could not be reached.
